# Acute Endoplasmic Reticulum Stress Suppresses Hepatic Gluconeogenesis by Stimulating MAPK Phosphatase 3 Degradation

**DOI:** 10.3390/ijms242115561

**Published:** 2023-10-25

**Authors:** Xiaohua Huang, Heng Zhu, Wei Lu, Lei Cao, Zhengfeng Fang, Lianqiang Che, Yan Lin, Shengyu Xu, Yong Zhuo, Lun Hua, Xuemei Jiang, Mengmeng Sun, De Wu, Bin Feng

**Affiliations:** 1 Animal Nutrition Institute, Sichuan Agricultural University, Chengdu 611130, China; hxh3028@163.com (X.H.); yxy0931120@163.com (H.Z.); lu_wei6565@163.com (W.L.); 13259374362@163.com (L.C.); zfang@sicau.edu.cn (Z.F.); chelianqiang@sicau.edu.cn (L.C.); linyan@sicau.edu.cn (Y.L.); shengyuxu@sicau.edu.cn (S.X.); zhuoyong@sicau.edu.cn (Y.Z.); hualun@sicau.edu.cn (L.H.); 71310@sicau.edu.cn (X.J.); 2 Key Laboratory of Animal Disease-Resistant Nutrition of Ministry of Education, Sichuan Agricultural University, Chengdu 611130, China; 3 College of Science, Sichuan Agricultural University, Chengdu 611130, China; 14391@sicau.edu.cn

**Keywords:** DILI, ER stress, PERK, MKP-3, gluconeogenesis

## Abstract

Drug-induced liver injury (DILI) is a widespread and harmful disease, and is closely linked to acute endoplasmic reticulum (ER) stress. Previous reports have shown that acute ER stress can suppress hepatic gluconeogenesis and even leads to hypoglycemia. However, the mechanism is still unclear. MAPK phosphatase 3 (MKP-3) is a positive regulator for gluconeogenesis. Thus, this study was conducted to investigate the role of MKP-3 in the suppression of gluconeogenesis by acute ER stress, as well as the regulatory role of acute ER stress on the expression of MKP-3. Results showed that acute ER stress induced by tunicamycin significantly suppressed gluconeogenesis in both hepatocytes and mouse liver, reduced glucose production level in hepatocytes, and decreased fasting blood glucose level in mice. Additionally, the protein level of MKP-3 was reduced by acute ER stress in both hepatocytes and mouse liver. *Mkp-3* deficiency eliminated the inhibitory effect of acute ER stress on gluconeogenesis in hepatocytes. Moreover, the reduction effect of acute ER stress on blood glucose level and hepatic glucose 6-phosphatase (*G6pc*) expression was not observed in the liver-specific *Mkp-3* knockout mice. Furthermore, activation of protein kinase R-like ER kinase (PERK) decreased the MKP-3 protein level, while inactivation of PERK abolished the reduction effect of acute ER stress on the MKP-3 protein level in hepatocytes. Taken together, our study suggested that acute ER stress could suppress hepatic gluconeogenesis by stimulating MKP-3 degradation via PERK, at least partially. Thus, MKP-3 might be a therapeutic target for DILI-related hypoglycemia.

## 1. Introduction

Drug-induced liver injury (DILI) is a widespread and harmful disease, which is usually caused by drugs or their metabolites, such as nonsteroidal anti-inflammatory drugs, anti-tuberculosis drugs, antiepileptic drugs and some traditional Chinese medicine (TCM) [1,2]. DILI is closely linked to endoplasmic reticulum (ER) stress [1,3]. ER stress can be classified into three types: acute, periodic, and chronic ER stress. DILI-related ER stress belongs to the acute type, which is typically induced by acute drugs and chemical treatments (e.g., acetaminophen, isoniazid, valproic acid, tunicamycin, dithiothreitol, calcium ionophores and saturated fatty acids) [4,5]. ER stress has three canonical signaling pathways; these are the protein kinase R-like ER kinase (PERK) pathway, the inositol-requiring enzyme 1 (IRE1) pathway, and the activating transcription factor 6 (ATF6) pathway. PERK has kinase activity, which phosphorylates and activates the eukaryotic translation initiation factor (eIF2α), and subsequently activates the expression of nuclear transcription factor ATF4. IRE1 has both endoribonuclease activity and kinase activity, which splices the mRNA of X-box binding protein 1 (sXBP1) and activates Jun N-terminal kinase (JNK), respectively. ATF6 induces the expression of ER chaperone, including 78 kDa glucose regulatory protein (GRP78) [6,7].

The liver is the main organ for the regulation of energy metabolism in the body, including lipid metabolism and glucose homeostasis. The liver regulates blood glucose level mainly through gluconeogenesis [8,9]. The process of gluconeogenesis involves a series of enzymatic reactions, with glucose 6-phosphatase (G6PC) and phosphoenolpyruvate carboxykinase (PEPCK) as the rate-limiting enzymes. The expression of these two genes can be regulated by peroxisome proliferator-activated receptor gamma coactivator 1 alpha (PGC1α) and forkhead box O1 (FOXO1) [9,10,11].

Studies have reported that chronic ER stress impairs insulin sensitivity, upregulates the expression of gluconeogenic genes *Pepck* and *G6pc*, and increases hepatic glucose production and blood glucose level in animals [12,13]. Conversely, acute ER stress can suppress the expression of gluconeogenic genes [14]. Tunicamycin (TM), which can induce acute ER stress in the liver, was proved to suppress gluconeogenesis and lead to hypoglycemia [15]. However, the mechanism by which acute ER stress suppresses gluconeogenesis is still unclear.

Our previous study found that MAPK phosphatase-3 (MKP-3) can positively regulate hepatic gluconeogenesis by dephosphorylating FOXO1 and promoting the expression of *Pgc1a* [16]. In addition, the protein level of MKP-3 could be down-regulated by hormones, such as insulin and leptin, in a phosphorylation–ubiquitination manner [17,18,19]. However, it is unknown whether MKP-3 was involved in the regulation of hepatic gluconeogenesis by acute ER stress. In the current study, we analyzed the effect of acute ER stress on gluconeogenesis with both in vitro and in vivo studies, and investigated the role of MKP-3 in the suppression of gluconeogenesis by acute ER stress, as well as exploring how acute ER stress regulated MKP-3 expression.

## 2. Results

### 2.1. Acute ER Stress Attenuated Hepatic Gluconeogenesis In Vitro

**Figure 1:**
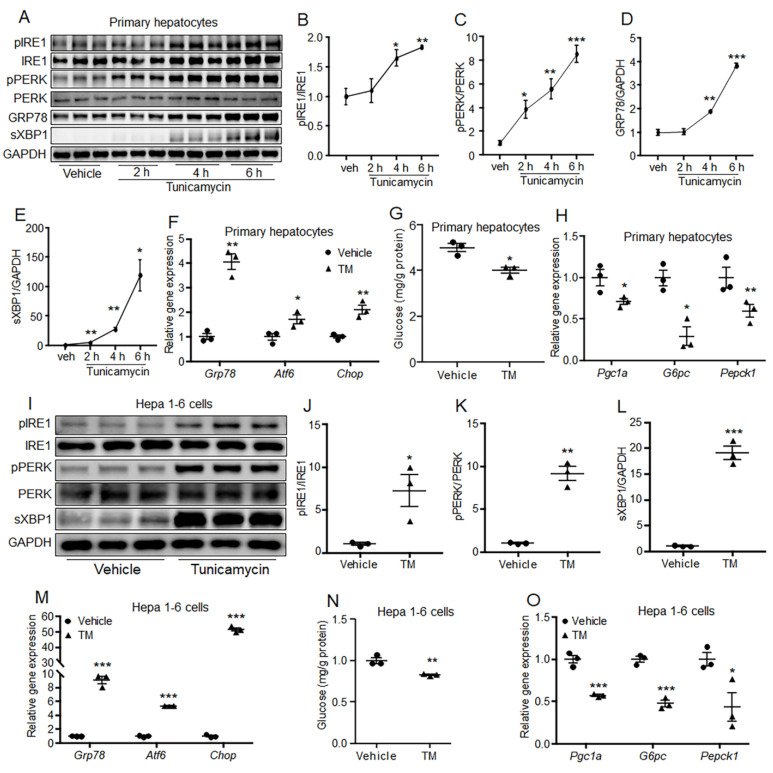
Acute ER stress suppressed gluconeogenesis in hepatocytes. (**A**–**E**) Primary mouse hepatocytes were treated with 1 μg/mL TM for the indicated time. The protein levels of GRP78 and sXBP1 and phosphorylation levels of IRE1 and PERK were detected using western blotting. (**F**–**H**) Primary mouse hepatocytes were treated with 1 μg/mL TM for 6 h; the mRNA levels of *Grp78*, *Atf6* and *Chop* (**F**), glucose production (**G**) and gene expression levels of *Pgc1a*, *G6pc* and *Pepck1* (**H**) were detected. (**I**–**L**) Hepa 1-6 cells were treated with 1 μg/mL TM for 6 h; the protein levels of GRP78 and sXBP1 and phosphorylation levels of IRE1 and PERK were detected. (**M**–**O**) Hepa 1-6 cells were treated with 1 μg/mL TM for 6 h; the mRNA levels of *Grp78*, *Atf6* and *Chop* (M), glucose production (**N**) and gene expression levels of *Pgc1a*, *G6pc* and *Pepck1* (**O**) were detected. *N* = 3 per group. * *p* < 0.05, ** *p* < 0.01, *** *p* < 0.001 as compared with the control group. Results represent 1 of 3 independently performed experiments. TM, tunicamycin.

The effect of acute ER stress on gluconeogenesis was firstly investigated in hepatocytes. Primary mouse hepatocytes and Hepa 1-6 cells were treated with TM to induce acute ER stress. Results showed that TM treatment over 4 h significantly induced phosphorylation levels of IRE1 and PERK, and protein levels of GRP78 and sXBP1 in primary hepatocytes, compared to the control treatment (Figure 1A–E). TM treatment for 6 h increased the mRNA levels of ER stress marker genes *Grp78*, *Atf6* and *Chop* in primary hepatocytes, compared with the control group (Figure 1F). Furthermore, glucose production and expression of gluconeogenic genes *Pepck1*, *G6pc*, and their regulatory gene, *Pgc1a,* were suppressed by TM treatment compared to the control group, in primary hepatocytes (Figure 1G,H). Moreover, similar results were observed in Hepa 1-6 cells (Figure 1I–O). These data indicate that acute ER stress can suppress gluconeogenesis in hepatocytes.

### 2.2. Acute ER Stress Suppressed Hepatic Gluconeogenesis In Mice

**Figure 2:**
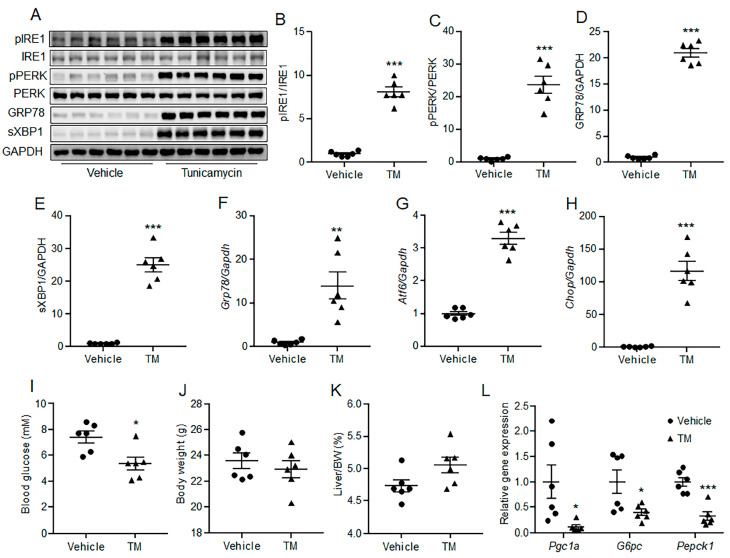
Acute ER stress suppressed hepatic gluconeogenesis in mice. C57BL/6N mice were injected intraperitoneally with 1 mg/kg TM or vehicle. (**A**–**E**) Phosphorylation levels of IRE1 and PERK, and protein levels of GRP78 and sXBP1 in the liver. (**F**–**H**) The mRNA levels of *Grp78* (**F**), *Atf6* (**G**) and *Chop* (**H**) in the liver. (**I**–**K**) Fasting blood glucose levels (**I**), body weight (**J**) and liver weight index (**K**) of the mice. (**L**) The expression levels of gluconeogenic genes in the liver. *N* = 6 for each group. * *p* < 0.05, ** *p* < 0.01, *** *p* < 0.001 as compared with the control group. TM, tunicamycin.

To further elucidate the effect of acute ER stress on in vivo hepatic gluconeogenesis, male C57BL/6N mice were injected intraperitoneally with 1 mg/kg TM or vehicle for 6 h. Results showed that TM administration induced ER stress in mouse liver (Figure 2A–H). The fasting blood glucose level was much lower in the TM group than that in the control group, though the body weight and liver weight were similar between the two groups (Figure 2I–K). Furthermore, hepatic gene expression levels of *Pgc1a*, *G6pc* and *Pepck1* were significantly down-regulated by acute ER stress, as compared with the control group (Figure 2L). These data suggest that acute ER stress can suppress hepatic gluconeogenesis in vivo.

### 2.3. Acute ER Stress Decreased MKP-3 Protein Level in Both Hepatocytes and Mouse Liver

**Figure 3:**
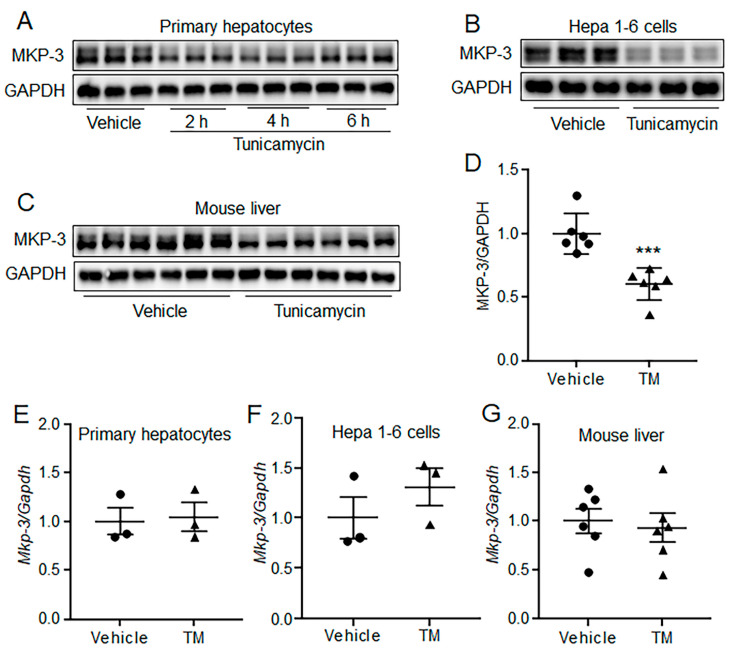
Acute ER stress decreased MKP-3 protein level in both hepatocytes and mouse liver. (**A**) MKP-3 protein levels in 1 μg/mL TM or vehicle-treated primary mouse hepatocytes. (**B**) MKP-3 protein levels in 1 μg/mL TM or vehicle-treated Hepa 1-6 cells. (**C**,**D**) MKP-3 protein levels in 1 mg/kg TM or vehicle-treated C57BL/6N mouse liver. (**E**,**F**) MKP-3 mRNA levels in 1 μg/mL TM or vehicle-treated primary mouse hepatocytes (**E**) and Hepa 1-6 cells (**F**). (**G**) MKP-3 mRNA levels in 1 mg/kg TM or vehicle-treated C57BL/6N mouse liver. *N* = 3 per treatment for cell studies, and *N* = 6 per group for mouse study. *** *p* < 0.001 as compared with the control group. Results for cell studies represent 1 of 3 independently performed experiments. TM, Tunicamycin.

Our previous studies have shown that MKP-3 could promote hepatic gluconeogenesis, and its protein level can be down-regulated by insulin and leptin in a ubiquitination manner [16,17,18]. Therefore, the expression of MKP-3 was determined in TM treated hepatocytes and mouse liver. Results showed that the protein level of MKP-3 was decreased by TM administration for 2 h or longer in primary hepatocytes, compared with the control group (Figure 3A). In addition, acute ER stress significantly decreased the MKP-3 protein level in Hepa 1-6 cells (Figure 3B). Furthermore, TM administration resulted in a significant decrease in MKP-3 protein level in mouse liver, as compared to the control treatment (Figure 3C,D). However, the mRNA level of MKP-3 was not changed by acute TM treatment neither in hepatocytes nor in mouse liver (Figure 3E–G). These data indicate that acute ER stress can decrease the protein level of MKP-3 in a post-transcriptional manner both in vivo and in vitro.

### 2.4. MKP-3 Was Involved in the Suppression of Gluconeogenesis by Acute ER Stress in Primary Hepatocytes

**Figure 4:**
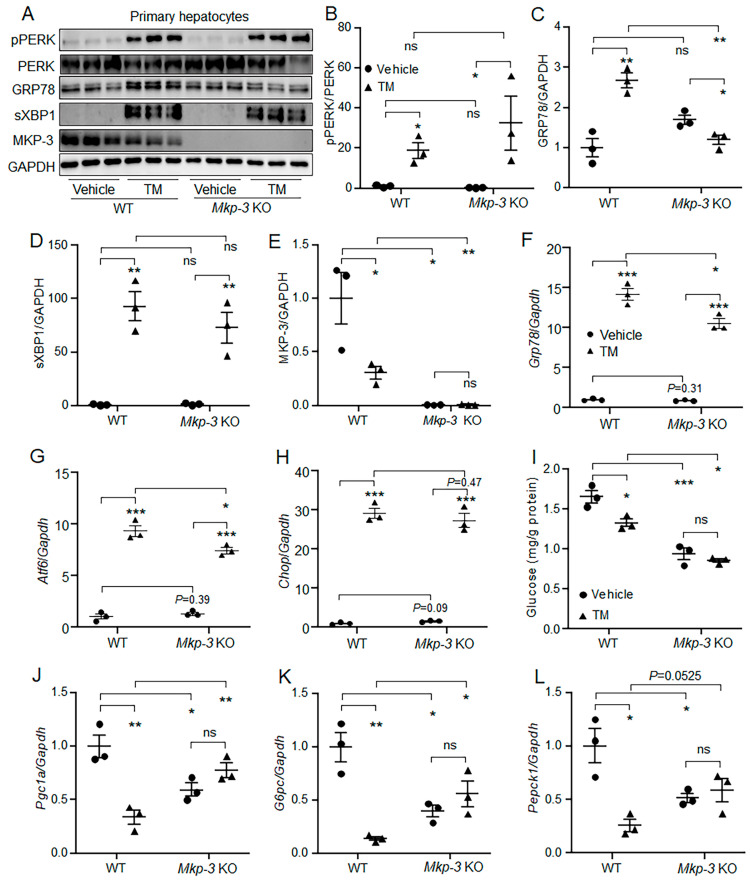
MKP-3 was involved in the suppression of gluconeogenesis by acute ER stress in primary mouse hepatocytes. *Mkp-3*-deficient primary mouse hepatocytes and wild-type primary mouse hepatocytes were treated with 1 μg/mL TM or vehicle. (**A**–**E**) The protein levels of pPERK, GRP78, sXBP1 and MKP-3 in the cells. (**F**–**H**) The gene expression levels of *Grp78* (**F**), *Atf6* (**G**), and *Chop* (**H**) in the cells. (**I**–**L**) The glucose production level (**I**) and gene expression levels of *Pgc1a* (**J**), *G6pc* (**K**) and *Pepck1* (**L**) in the cells. *N* = 3 per group. * *p* < 0.05, ** *p* < 0.01, *** *p* < 0.001 as indicated. ns, no significance. Results represent 1 of 3 independently performed experiments. TM, Tunicamycin.

The role of MKP-3 in the suppression of gluconeogenesis by acute ER stress was then investigated in *Mkp-3* knockout (KO) primary mouse hepatocytes. Results showed that though TM induced acute ER stress signaling and the mRNA level of *Grp78* in both wild-type (WT) and *Mkp-3*-deficient primary hepatocytes, the protein level of GRP78 was not changed by TM treatment in *Mkp-3* KO hepatocytes, as compared to the control treatment (Figure 4A–H). In addition, *Mkp-3* deficiency blocked the suppression effect of acute ER stress on glucose production and gluconeogenic gene expression in primary hepatocytes (Figure 4I–L). These data indicate that MKP-3 is involved in the suppression of gluconeogenesis by acute ER stress in primary hepatocytes.

### 2.5. MKP-3 Was Involved in the Suppression of Hepatic Gluconeogenesis by Acute ER Stress in Mouse

**Figure 5:**
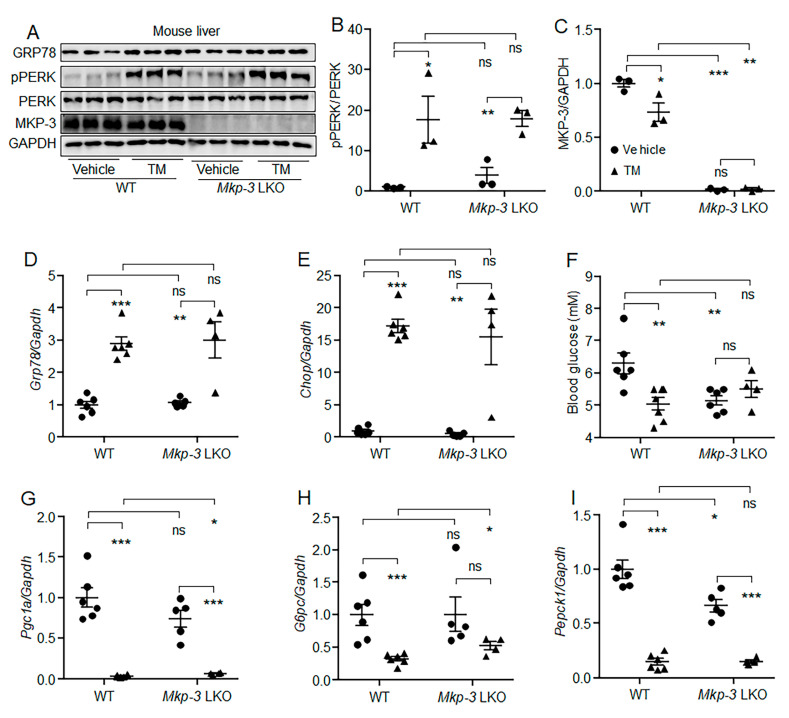
MKP-3 was involved in the suppression of hepatic gluconeogenesis by acute ER stress in mice. Liver-specific *Mkp-3* knockout (*Mkp-3* LKO) mice and wild-type (WT) littermates were injected intraperitoneally with 1 mg/kg TM or vehicle. (**A**–**C**) Phosphorylation level of PERK, and protein levels of GRP78 and MKP-3 in the liver. (**D**,**E**) The mRNA levels of *Grp78* (**D**) and *Chop* (**E**) in the liver. (**F**) Fasting blood glucose levels of mice. (**G**–**I**) The mRNA levels of *Pgc1a* (**G**), *G6pc* (**H**) and *Pepck1* (**I**) in the liver. *N* = 4–6 per group. * *p* < 0.05, ** *p* < 0.01, *** *p* < 0.001 as indicated. ns, no significance. TM, Tunicamycin.

The role of MKP-3 in the suppression of hepatic gluconeogenesis by acute ER stress was then investigated in mice. Results showed that although TM induced acute ER stress in the liver of both WT and liver-specific *Mkp-3* knockout (*Mkp-3* LKO) mice (Figure 5A–E), the reduction effect of TM on blood glucose level and gene expression of hepatic *G6pc* was not observed in *Mkp-3*-deficient mice, while the expression of *Pgc1a* and *Pepck1* were still suppressed by TM in the liver of *Mkp-3* LKO mice (Figure 5F–I). These data indicate that MKP-3 might be involved in the suppression of hepatic gluconeogenesis by acute ER stress in mice, at least partially.

### 2.6. IRE1 Was Not Needed for the Reduction of MKP-3 Protein Level by Acute ER Stress

**Figure 6:**
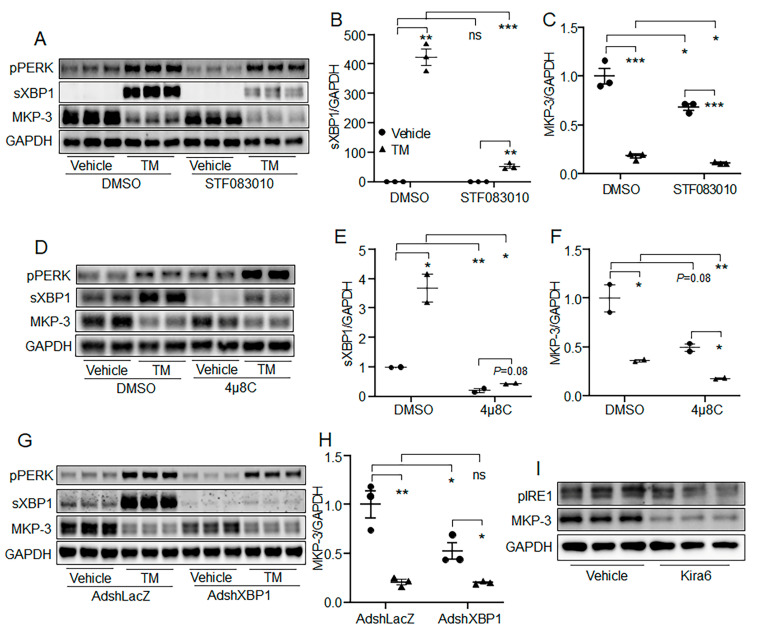
IRE1 was not necessary for the reduction of MKP-3 by acute ER stress. (**A**–**C**) Primary mouse hepatocytes were pretreated with 50 μM STF083010 or vehicle for 24 h, followed by treatment with TM plus STF083010 or vehicle for 6 h. The protein levels of pPERK, sXBP1 and MKP-3 were detected. (**D**–**F**) Primary mouse hepatocytes were pretreated with 5 μM 4μ8C or vehicle for 24 h, followed by treatment with TM plus 4μ8C or vehicle for 6 h. The protein levels of pPERK, sXBP1 and MKP-3 were detected. (**G**,**H**) Primary mouse hepatocytes were infected with AdshLacZ or AdshXBP1. Forty-eight hours later, cells were treated with TM or vehicle for 6 h. The protein levels of pPERK, XBP1 and MKP-3 were detected. (**I**) Primary mouse hepatocytes were treated with Kira6 or vehicle for 6 h. The protein levels of pIRE1 and MKP-3 were detected. * *p* < 0.05, ** *p* < 0.01, *** *p* < 0.001 as indicated. ns, no significance. Results represent 1 of 3 independently performed experiments. TM, Tunicamycin.

ER stress induces three canonical signaling pathways: the PERK/eIF2α pathway, IRE1/sXBP1 pathway, and ATF6 pathway. Of them, ATF6 is a transcription factor, while PERK is a kinase, and IRE1 has both endonuclease activity and kinase activity. Acute ER stress only decreased the protein level of MKP-3, but not its mRNA level. And, previous studies have reported that MKP-3 protein could be degraded in a phosphorylation–ubiquitination manner. Thus, we investigated whether acute ER stress reduced the MKP-3 protein level through the IRE1 pathway. Results showed that the reduction effect of acute ER stress on MKP-3 could not be blocked by the IRE1 endonuclease inhibitor STF083010 or 4μ8C, but was promoted by them (Figure 6A–F). In addition, knock-down of *Xbp1* using its shRNA in hepatocytes got similar results to the IRE1 inhibitor study (Figure 6G,H). Furthermore, Kira6, the inhibitor for IRE1 kinase, decreased the MKP-3 protein level in hepatocytes (Figure 6I). These results indicate that acute ER stress-promoted MKP-3 protein degradation is likely independent of the IRE1 pathway.

### 2.7. PERK Was Involved in the Reduction of MKP-3 Protein Level by Acute ER Stress

**Figure 7:**
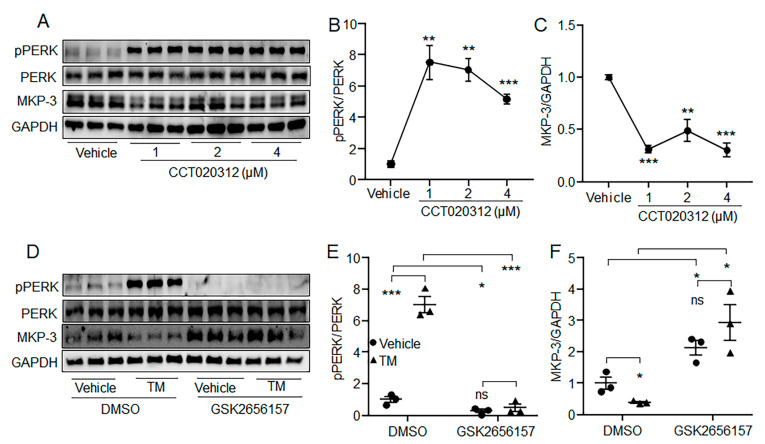
PERK was needed for the reduction of MKP-3 protein level by acute ER stress in hepatocytes. (**A**–**C**) Primary mouse hepatocytes were treated with CCT020312 at the indicated doses for 2 h. Phosphorylation level of PERK and the protein level of MKP-3 were detected. (**D**–**F**) Primary mouse hepatocytes were pretreated with 1 μM GSK2656157 or vehicle for 24 h, followed by treatment with TM plus GSK2656157 or vehicle for 6 h. Phosphorylation level of PERK and the protein level of MKP-3 were detected. *N* = 3 for each group. * *p* < 0.05, ** *p* < 0.01, *** *p* < 0.001 as indicated. ns, no significance. Results represent 1 of 3 independently performed experiments. TM, Tunicamycin.

We then investigated whether PERK was involved in the reduction of the MKP-3 protein level by acute ER stress. Results showed that the MKP-3 protein level could be suppressed by PERK activator CCT020312 (Figure 7A–C). Furthermore, the inhibitor of PERK GSK2656157 restored the MKP-3 protein level that was reduced by acute TM treatment (Figure 7D–F). These results suggest that acute ER stress might decrease MKP-3 protein level via PERK pathway in hepatocytes, at least partially.

## 3. Discussion

Drug-induced liver injury, which is closely linked to acute endoplasmic reticulum (ER) stress, is a widespread disease, and might induce hypoglycemia [1,20,21]. Here we showed that activation of acute ER stress by tunicamycin suppressed gluconeogenesis in both hepatocytes and mouse liver, as well as reducing glucose production in hepatocytes and fasting blood level in mice. In addition, the protein level of MKP-3, a positive regulator for gluconeogenesis, was decreased by acute ER stress both in vitro and in vivo. Furthermore, *Mkp-3* knockout abolished the suppression effect of acute ER stress on gluconeogenesis both in hepatocytes and mouse liver. Thus, MKP-3 might be a potential therapeutic target in drug-induced hypoglycemia.

ER stress can be classified into three types: acute, periodic, and chronic [5]. DILI-related ER stress belongs to the acute one, which is typically induced by acute drugs and chemical treatments [3]. TM, which has a potential therapeutic effect on cancer treatment [22], was typically used to induce acute ER stress for both in vivo and in vitro studies [23,24]. In the current study, our results showed that TM treatment significantly increased the mRNA levels of *Grp78*, *Atf6* and *Chop*, and enhanced phosphorylation levels of IRE1 and PERK and protein levels of sXBP1 and GRP78. These data suggest that acute ER stress was induced in our study. It is interesting that the protein level of GRP78 was not induced by TM in *Mkp-3* KO hepatocytes, while the ER stress signaling and gene expression of *Grp78* was induced. GRP78 is an ER chaperone, which binds to unfolded or misfolded polypeptide chains and/or unassembled multi-subunit proteins, leading to the release and, consequently, the activation of the ER stress sensors [25]. Thus, our data suggest that MKP-3 might be involved in the unfold protein response (UPR) in hepatocytes. Alternatively, there might be a post-transcriptional regulation of GRP78 by MKP-3. However, the GRP78 protein level was induced by TM in the liver of *Mkp-3* LKO mice. These might because the regulatory effect MKP-3 on GRP78 was eliminated by some in vivo hormones. This will be further elucidated in future studies.

It has been reported that hepatic glucose homeostasis can be disrupted by ER stress, which is one of the reasons for metabolic diseases [5,15]. Here, we showed that short-time TM administration induced acute ER stress, decreased glucose production in primary hepatocytes and blood glucose level in mice, and suppressed the expression of hepatic gluconeogenic genes. Similarly, Seo et al. suggested that TM treatment for 16 h could decrease blood glucose levels and the expression of *Pepck* and *G6pc* in mice [14]. Wang et al. also suggested that TM treatment for 10 h decreased blood glucose level and suppressed the expression of *Pepck* and *G6pc* in mice [15]. However, our previous study showed that 24 h TM treatment decreased blood level, but did not change the expression and activities of G6PC and PEPCK in mice [23]. This might be because different times of TM treatment induced different types of ER stress. Though the effect of chronic ER stress on gluconeogenesis has been studied in diet-induced obese mice [26], the effect of long-time low-dose TM treatment will be investigated in future studies.

Here, we observed that the protein level of MKP-3 was decreased by short-time TM treatment both in hepatocytes and mouse liver. MKP-3 is considered a novel molecular for the regulation of hepatic glucose homeostasis. Previous reports have shown that MKP-3 stimulates hepatic gluconeogenesis by promoting the expression of *Pgc1a* and dephosphorylating FOXO1 [16,27]. We have previously shown that MKP-3 was involved in the suppression of gluconeogenesis by hepatic leptin signaling [18]. Here, we found that *Mkp-3* deficiency eliminated the suppression effect of acute ER stress on gluconeogenesis in primary hepatocytes. In the *Mkp-3* LKO mice, TM did not change the blood glucose level and expression of *G6pc*. These data suggest that MKP-3 might be also involved in the suppression of gluconeogenesis by acute ER stress, at least partially. However, TM still decreased the expression of *Pgc1a* and *Pepck1* in the liver of *Mkp-3* LKO mice. This might be because there was a compensatory increase in any other phosphatases in *Mkp-3* LKO mice, like DUSP4 [28]. And these phosphatases regulated the expression of *Pgc1a* and *Pepck1*. This hypothesis will be elucidated in future studies. 

MKP-3 protein can be degraded by several hormones in a kinase–ubiquitination manner [17,18,29,30]. Bermudez et al. suggested that serum growth factor induced the degradation of MKP-3 through the mTOR pathway [29]. Feng et al. suggested that insulin, the major hormone suppressing gluconeogenesis, promoted the degradation of MKP-3 protein through the ERK pathway in hepatocytes [17]. In addition, our previous report showed that the adipokine leptin decreased the MKP-3 protein level through STAT3 in hepatocytes [18]. The current study showed that the protein level of MKP-3 was decreased by acute TM treatment, but no change in *Mkp-3* mRNA level was observed. These data suggested that acute ER stress might suppress MKP-3 expression in a post-translational manner. Further study will be carried out to confirm whether Serine to Alanine mutation of MKP-3 at Ser159 and Ser197 resists TM-induced degradation.

IRE1/sXBP1 and PERK/eIF2α are two typic pathways in ER stress. IRE1 has both endoribonuclease activity and kinase activity [25]. Activated IRE1 can bind to the adaptor protein tumor-necrosis factor-α (TNF-α)-receptor-associated factor 2 (TRAF2), and then induce the phosphorylation and degradation of the inhibitor of NF-κB (IκB) [31,32]. PERK can be activated by ER stress-induced oligomerization, and subsequently phosphorylates the eukaryotic translation initiation factor 2 (eIF2α) [25,33]. Thus, the roles of IRE1 and PERK in the reduction of MKP-3 protein level by TM treatment were investigated. Our data showed that inhibition of the endoribonuclease activity of IRE1 or knockdown of XBP1 could not reverse the protein level of MKP-3 that was reduced by acute TM treatment. Furthermore, the inhibitor for IRE1 kinase activity even decreased the protein level of MKP-3. These results suggest that acute ER stress-induced MKP-3 degradation might be independent of IRE1.

Further study showed that the activation of PERK decreased MKP-3 protein level in hepatocytes, while the inhibition of PERK restored the MKP-3 protein level that was decreased by acute TM treatment. This result indicates that acute ER stress might decrease MKP-3 protein level through PERK, at least partly. PERK can phosphorylate heterogeneous nuclear ribonucleoprotein A1 (HNRNPA1) at Thr51, causing it to be degraded by the proteasome [34]. MKP-3 can be phosphorylated at Ser159 and Ser197, and then degraded in a ubiquitination manner [30]. Thus, PERK might regulate the protein level of MKP-3 by inducing its phosphorylation Ser159 and Ser197. Further study will be conducted to confirm this hypothesis.

## 4. Materials and Methods

### 4.1. Animal Studies

All animal procedures were reviewed and approved by the Animal Ethical and Welfare Committee of Sichuan Agricultural University (20190122), and were carried out in accordance with the Guide for the Care and Use of Laboratory Animals (National Research Council, Bethesda, MD, USA). Four-week-old male mice (C57BL/6N) were obtained from Vital River Laboratory Animal Technology Co. Ltd. (Beijing, China), and were kept in a pathogen free room at 22 °C and a 60% stable temperature and humidity. When they reached 8 weeks of age, twelve mice were randomly divided into two groups, and they were injected intraperitoneally with 1 mg/kg tunicamycin (TM, Sigma, St. Louis, MO, USA) or vehicle. The body weight and blood glucose level (blood glucose strips (5D-2) were purchased from Beijingyicheng, Beijing, China) under fasted state were measured 6 h after injection. The mice were then euthanized using carbon dioxide, followed by cervical dislocation. Liver was collected for further analysis.

The liver-specific *Mkp-3* knockout (*Mkp-3* LKO) mice were generated by cross mating *Mkp-3*^loxp/loxp^ mice (Cyagen Biosciences, Guangzhou, China) with albumin-Cre mice (Jackson Laboratory, Bar Harbor, ME, USA). The *Mkp-3*^loxp/loxp^ littermate mice were used as the control group. Twelve-week-old male *Mkp-3* LKO mice and their corresponding control mice were injected intraperitoneally with 1 mg/kg TM or vehicle. Liver samples were collected 6 h after injection under fasting state.

### 4.2. Cell Culture and Treatment

Hepa 1-6 hepatoma cells (provided by Dr. Gökhan Hotamisligil, Harvard School of Public Health, Boston, MA, USA) and primary mouse hepatocytes were cultured in Dulbecco’s modified Eagle medium (DMEM) containing 10% fetal bovine serum (FBS), 100 U/mL penicillin and 100 μg/mL streptomycin (Gibco, Shanghai, China) at 37 °C with 5% CO_2_. Primary mouse hepatocytes were isolated by infusing mouse liver with collagenase as previously reported [35]. 

Hepa 1-6 cells and primary mouse hepatocytes were seeded in a 12-well plate at a density of 4 × 10^5^ cells/well. Cells were treated with 1 μg/mL TM [36,37] or vehicle for 6 h after an overnight incubation with serum-free DMEM. For the inhibitors study, hepatocytes were pretreated with 50 μM STF083010 (inhibitor for IRE1, Selleck Chemical, Houston, TX, USA), 5 μM 4μ8C (inhibitor for IRE1, Selleck Chemical, Houston, TX, USA) or 1 μM GSK2656157 (inhibitor for PERK, Selleck Chemical, Houston, TX, USA) for 24 h, followed by TM plus inhibitor treatment for 6 h. For PERK activation study, primary mouse hepatocytes were treated with 1, 2, or 4 μM CCT020312 (Selleck Chemical, Houston, TX, USA) for 2 h. For IRE1 activation study, primary mouse hepatocytes were treated with 5 μM Kira6 (Selleck Chemical, Houston, TX, USA) or vehicle for 6 h.

### 4.3. Glucose Output Assay

The glucose output assay was performed as previously reported [18]. Hepatocytes were washed 3 times with phosphate-buffered saline (PBS) and were incubated in serum-free DMEM containing 0.5 mM 8-bromo-cyclic adenosine monophosphate (cAMP, Sigma, St. Louis, MO, USA) and 1 μg/mL TM or vehicle for 5 h. Cells were then incubated in 0.5 mL/well of phenol red-free, glucose-free DMEM (Sigma, St. Louis, MO, USA) containing 2 mM pyruvate (Sigma, St. Louis, MO, USA), 20 mM lactate (Sigma, St. Louis, MO, USA), and 1 mM 8-bromo-cAMP, with 1 μg/mL TM or vehicle. Medium was collected 3 h later and subjected to glucose measurement using the Glucose Assay Kit (Sigma, St. Louis, MO, USA). Cells were lysed and the protein concentration was measured. The glucose production was normalized with cellular protein content. 

### 4.4. RNA Extraction and Real-Time PCR

RNA extraction and real-time PCR were performed as previously reported [18]. RNA was extract with Trizol Reagent (Sigma, St. Louis, MO, USA). cDNA was synthetized with a reverse transcription PCR kit (Thermofisher Scientific, Shanghai, China). Real-time PCR was performed on a quantitative-PCR machine (7900HT, ABI, Carlsbad, CA, USA) with Power SYBR Green RT-PCR reagents (BioRad, Hercules, CA, USA). The sequence of primers is listed in Table 1.

### 4.5. Western Blot Analysis

Total proteins were extracted from liver tissues and hepatocytes using cell lysis buffer, and the aim proteins were detected with western blotting using specific antibodies as described previously [18]. The anti-MKP-3 (sc-377070) and anti-IRE1 (sc-390960) antibodies were purchased from Santa Cruz Biotechnology (Santa Cruz, Dallas, TX, USA), anti-phospho-IRE1 (Ser724, ab48187) antibody was from Abcam (Cambridge, MA, USA), anti-sXBP1 (#83418), anti-phospho-PERK (Thr980, #3179s) and anti-PERK (#4970) antibodies were purchased from Cell Signaling Technology (Danvers, MA, USA), anti-GRP78 (1157-1-AP) antibody was from proteintech (Wuhan, China), and anti-GAPDH (abs132994) antibody was from Absin (Shanghai, China).

### 4.6. Statistical Analysis

Data were analyzed with SAS 9.3 software (Cary, NC, USA). An independent *t*-test was used to compare the difference between two groups. The results were presented as mean ± SE. Statistical significance was determined at *p* < 0.05. All cell studies were repeated at least three times.

## 5. Conclusions

Acute ER stress could suppress hepatic gluconeogenesis by stimulating MKP-3 degradation via PERK, at least partially. Thus, MKP-3 is a potential therapeutic target for DILI-related hypoglycemia.

## Figures and Tables

**Table 1:** Primers for Real-Time quantitative PCR.

Gene Name	Forward Primer	Reverse Primer
*Gapdh*	AGGTCGGTGTGAACGGATTTG	TGTAGACCATGTAGTTGAGGTCA
*Pgc1a*	TATGGAGTGACATAGAGTGTGCT	CCACTTCAATCCACCCAGAAAG
*Pepck1*	CGCTGGATGTCGGAAGAGG	GGCGAGTCTGTCAGTTCAATAC
*G6pc*	CGACTCGCTATCTCCAAGTGA	GTTGAACCAGTCTCCGACCA
*Grp78*	ATCAGGGCAACCGCATCAC	TGATGTCCTGCTGCACCGAA
*Atf6*	CGGTCCACAGACTCGTGTTC	GCTGTCGCCATATAAGGAAAGG
*Chop*	CACGCACATCCCAAAGCC	GGGCACTGACCACTCTGTT
*Mkp3*	TGCGGGCGAGTTCAAATACA	AGCAATGCACCAGGACACCA

## Data Availability

The data presented in this study are available on request from the corresponding author.

## References

[1] Andrade R.J., Chalasani N., Bjornsson E.S., Suzuki A., Kullak-Ublick G.A., Watkins P.B., Devarbhavi H., Merz M., Lucena M.I., Kaplowitz N. (2019). Drug-induced liver injury. Nat. Rev. Dis. Primers.

[2] Hoofnagle J.H., Björnsson E.S. (2019). Drug-Induced Liver Injury—Types and Phenotypes. N. Engl. J. Med..

[3] Pu S., Pan Y., Zhang Q., You T., Yue T., Zhang Y., Wang M. (2023). Endoplasmic Reticulum Stress and Mitochondrial Stress in Drug-Induced Liver Injury. Molecules.

[4] Iorga A., Dara L., Kaplowitz N. (2017). Drug-Induced Liver Injury: Cascade of Events Leading to Cell Death, Apoptosis or Necrosis. Int. J. Mol. Sci..

[5] Fu S., Watkins S.M., Hotamisligil G.S. (2012). The Role of Endoplasmic Reticulum in Hepatic Lipid Homeostasis and Stress Signaling. Cell Metab..

[6] Malhi H., Kaufman R.J. (2011). Endoplasmic reticulum stress in liver disease. J. Hepatol..

[7] Hotamisligil G.S. (2010). Endoplasmic reticulum stress and the inflammatory basis of metabolic disease. Cell.

[8] Basu R., Chandramouli V., Dicke B., Landau B., Rizza R. (2005). Obesity and Type 2 Diabetes Impair Insulin-Induced Suppression of Glycogenolysis as Well as Gluconeogenesis. Diabetes.

[9] Puigserver P., Rhee J., Donovan J., Walkey C.J., Yoon J.C., Oriente F., Kitamura Y., Altomonte J., Dong H., Accili D. (2003). Insulin-regulated hepatic gluconeogenesis through FOXO1–PGC-1α interaction. Nature.

[10] Smith G.C., Turner N. (2017). FOXO1 is the headline Akt regulating hepatic glucose metabolism. Endocrinology.

[11] Sharabi K., Lin H., Tavares C.D., Dominy J.E., Camporez J.P., Perry R.J., Schilling R., Rines A.K., Lee J., Hickey M. (2017). Selective Chemical Inhibition of PGC-1α Gluconeogenic Activity Ameliorates Type 2 Diabetes. Cell.

[12] Wagner M., Moore D.D. (2011). Endoplasmic reticulum stress and glucose homeostasis. Curr. Opin. Clin. Nutr. Metab. Care.

[13] Kimura K., Yamada T., Matsumoto M., Kido Y., Hosooka T., Asahara S.-I., Matsuda T., Ota T., Watanabe H., Sai Y. (2011). Endoplasmic reticulum stress inhibits STAT3-dependent suppression of hepatic gluconeogenesis via dephosphorylation and deacetylation. Diabetes.

[14] Seo H.Y., Kim M.K., Min A.K., Kim H.S., Ryu S.Y., Kim N.K., Lee K.M., Kim H.J., Choi H.S., Lee K.U. (2010). Endoplasmic reticulum stress-induced activation of activating transcription factor 6 decreases cAMP-stimulated hepatic gluconeogenesis via inhibition of CREB. Endocrinology.

[15] Wang Y., Vera L., Fischer W.H., Montminy M. (2009). The CREB coactivator CRTC2 links hepatic ER stress and fasting gluconeogenesis. Nature.

[16] Wu Z., Jiao P., Huang X., Feng B., Feng Y., Yang S., Hwang P., Du J., Nie Y., Xiao G. (2010). MAPK phosphatase-3 promotes hepatic gluconeogenesis through dephosphorylation of forkhead box O1 in mice. J. Clin. Investig..

[17] Feng B., Jiao P., Yang Z., Xu H. (2012). MEK/ERK pathway mediates insulin-promoted degradation of MKP-3 protein in liver cells. Mol. Cell Endocrinol..

[18] Huang X., He Q., Zhu H., Fang Z., Che L., Lin Y., Xu S., Zhuo Y., Hua L., Wang J. (2022). Hepatic Leptin Signaling Improves Hyperglycemia by Stimulating MAPK Phosphatase-3 Protein Degradation via STAT3. Cell Mol. Gastroenterol. Hepatol..

[19] Jurek A., Amagasaki K., Gembarska A., Heldin C.-H., Lennartsson J. (2009). Negative and positive regulation of MAPK phosphatase 3 controls platelet-derived growth factor-induced Erk activation. J. Biol. Chem..

[20] Chan J.C.N., Cockram C.S., Critchley J.A.J.H. (1996). Drug-induced disorders of glucose metabolism. Drug Saf..

[21] Douillard C., Jannin A., Vantyghem M.-C. (2020). Rare causes of hypoglycemia in adults. Ann. d’Endocrinologie.

[22] Choi Y.J., Lee J.E., Ji H.D., Lee B.R., Lee S.B., Kim K.S., Lee I.K., Chin J., Cho S.J., Lee J. (2021). Tunicamycin as a Novel Redifferentiation Agent in Radioiodine Therapy for Anaplastic Thyroid Cancer. Int. J. Mol. Sci..

[23] Feng B., Huang X., Jiang D., Hua L., Zhuo Y., Wu D. (2017). Endoplasmic Reticulum Stress Inducer Tunicamycin Alters Hepatic Energy Homeostasis in Mice. Int. J. Mol. Sci..

[24] Song Q., Chen Y., Wang J., Hao L., Huang C., Griffiths A., Sun Z., Zhou Z., Song Z. (2020). ER stress-induced upregulation of NNMT contributes to alcohol-related fatty liver development. J. Hepatol..

[25] Zhang K., Kaufman R.J. (2008). From endoplasmic-reticulum stress to the inflammatory response. Nature.

[26] Zhang Z., Wang X., Zheng G., Shan Q., Lu J., Fan S., Sun C., Wu D., Zhang C., Su W. (2016). Troxerutin Attenuates Enhancement of Hepatic Gluconeogenesis by Inhibiting NOD Activation-Mediated Inflammation in High-Fat Diet-Treated Mice. Int. J. Mol. Sci..

[27] Xu H., Yang Q., Shen M., Huang X., Dembski M., Gimeno R., Tartaglia L.A., Kapeller R., Wu Z. (2005). Dual specificity MAPK phosphatase 3 activates PEPCK gene transcription and increases gluconeogenesis in rat hepatoma cells. J. Biol. Chem..

[28] Ito T., Young M.J., Li R., Jain S., Wernitznig A., Krill-Burger J.M., Lemke C.T., Monducci D., Rodriguez D.J., Chang L. (2021). Paralog knockout profiling identifies DUSP4 and DUSP6 as a digenic dependence in MAPK pathway-driven cancers. Nat. Genet..

[29] Bermudez O., Marchetti S., Pages G., Gimond C. (2008). Post-translational regulation of the ERK phosphatase DUSP6/MKP3 by the mTOR pathway. Oncogene.

[30] Marchetti S., Gimond C., Chambard J.C., Touboul T., Roux D., Pouyssegur J., Pages G. (2005). Extracellular signal-regulated kinases phosphorylate mitogen-activated protein kinase phosphatase 3/DUSP6 at serines 159 and 197, two sites critical for its proteasomal degradation. Mol. Cell Biol..

[31] Urano F., Wang X., Bertolotti A., Zhang Y., Chung P., Harding H.P., Ron D. (2000). Coupling of stress in the ER to activation of JNK protein kinases by transmembrane protein kinase IRE1. Science.

[32] Hu P., Han Z., Couvillon A.D., Kaufman R.J., Exton J.H. (2006). Autocrine tumor necrosis factor alpha links endoplasmic reticulum stress to the membrane death receptor pathway through IRE1alpha-mediated NF-kappaB activation and down-regulation of TRAF2 expression. Mol. Cell Biol..

[33] Ajoolabady A., Kaplowitz N., Lebeaupin C., Kroemer G., Kaufman R.J., Malhi H., Ren J. (2023). Endoplasmic reticulum stress in liver diseases. Hepatology.

[34] Koo J.H., Lee H.J., Kim W., Kim S.G. (2016). Endoplasmic Reticulum Stress in Hepatic Stellate Cells Promotes Liver Fibrosis via PERK-Mediated Degradation of HNRNPA1 and Up-regulation of SMAD2. Gastroenterology.

[35] Feng B., Jiao P., Helou Y., Li Y., He Q., Walters M.S., Salomon A., Xu H. (2014). Mitogen-activated protein kinase phosphatase 3 (MKP-3)-deficient mice are resistant to diet-induced obesity. Diabetes.

[36] Lee S.Y., Hong I.K., Kim B.R., Shim S.M., Lee J.S., Lee H.-Y., Soo Choi C., Kim B.K., Park T.S. (2015). Activation of sphingosine kinase 2 by endoplasmic reticulum stress ameliorates hepatic steatosis and insulin resistance in mice. Hepatology.

[37] Achard C.S., Laybutt D.R. (2012). Lipid-induced endoplasmic reticulum stress in liver cells results in two distinct outcomes: Adaptation with enhanced insulin signaling or insulin resistance. Endocrinology.

